# **Milk of livestock as a possible transmission route of** ***Helicobacter pylori*** **infection**

**Published:** 2015

**Authors:** Ramin Talaei, Negar Souod, Hassan Momtaz, Hossein Dabiri

**Affiliations:** 1 *Shahid Modaress Hospital, Faculty of Medicine, Shahid Beheshti University of Medical Sciences, Tehran, Iran *; 2 *Young Researchers and Elite Club, Islamic Azad University, Central Tehran Branch, Tehran, Iran *; 3 *Department of Microbiology, Faculty of Veterinary Medicine, Islamic Azad University, ShahreKord Branch, ShahreKord, Iran *; 4 *Department of Clinical Microbiology, Faculty of Medicine, Shahid Beheshti University of Medical Sciences, Tehran, Iran *

**Keywords:** *Helicobacter pylori*, Milk, Ruminant, Human, Virulence factors

## Abstract

**Aim:**

The current investigation aimed to evaluate ruminant raw milk as a reservoir source of *Helicobacter pylori *and analyze the diversity of *cagA* and *vacA* genotypes as *H. pylori *virulence factors to find any relationship between these genotypes in human and animal *H. pylori *strains.

**Background:**

The way of transmission of *Helicobacter* *pylori* as one of the most controversial bacteria in the world, which colonizes the human gastric tissue and is responsible for several gastric diseases is still unknown. The possibility of zoonotic transmission of *H. pylori *is feasible, but is not proven in ruminant reservoirs.

**Methods:**

Overall 210 cows, sheep, goats, camels and buffalos’ raw milk samples and 100 human gastric biopsies were collected in this survey. We applied PCR assays to identify *H. pylori*, *vacA* and *cagA* genes. Statistical tests were applied for data analysis.

**Results:**

Totally 12(16%) cow, 8(13.79%) sheep, 2 (4.76%) goat, 2(13.33%), buffalo 4(20%) and 82 (82%) of human specimens were confirmed to be *H. pylori *positive. Among which s1a/m2 genotype was more frequent in isolated *H. pylori *strains and statistically significant between strains. Based on statistical analyses the s1b allele of sheep had a significant association with human strains.

**Conclusion:**

The current survey was prompted by our previous report. According to both results we can conclude that sheep may act as a reservoir for *H. pylori *and transmit this bacterium to human via its milk. Extended assessments in other geographical regions and other animals are recommended.

## Introduction

 More than half of the world’s population is infected with *Helicobacter pylori*, a Gram-negative bacterium etiologically associated with gastrointestinal diseases, gastric adenocarcinoma and gastric B-cell lymphoma (1). 

There are reports describing significant differences in the prevalence of this bacterium between variant geographical regions and ethnics of each population (2). The exact routes by which humans acquire *H. pylori *infection are unknown (3). The available data suggest that the transmission of this bacterium occurs by person-to-person or by environmental sources such as water and milk to human. Recently zoonotic transmissions are considered as main routs of infection (4). Reported vectors are cows, sheep, cockroaches, houseflies as well as domestic pets (4). Several studies have shown higher levels of antibody in veterinarians, butchers and slaughters suggesting that ruminants might be a source of infection (3).

According to previous studies, the variety of clinical manifestations in the patients who suffer from *H. pylori *infections depends on multiple host and bacterial factors including host genetic, host immune response, bacterial load and virulence factors (5). One of the most extensively studied toxins which secreted by *H. pylori *is vacuolating cytotoxin A (VacA). As we mentioned in previous investigations, specific allelic diversity exists in domains of this gene: signal region (s1a, s1b, s1c and s2), the intermediate (i1, i2 and i3) and mid region (m1a, m1b and m2) (6,7). One other virulence factor is cytotoxin-associated gene A (*cagA*), which is a gene of pathogenicity island of *H. pylori *and can be a predictive marker for the bacterium pathogenicity (8). *H. pylori *strains can be divided into two groups: *cag* positive and *cag* negative. According to previous studies, *cag* positive strains are more prevalent in patients with more severe clinical symptoms (9).

Milk is a beneficial drink for the health of the human body, and most of the people include it at least once a day in their food chain. Besides appropriate conditions of milk provides an opportunity for *H. pylori *transition to human being. This study was prompted by our previous observation, in which we concluded that sheep might act as a reservoir for *H. pylori *and somehow share the ancestral host of this bacterium with human. In the current study we investigated the prevalence of *H. pylori *in the milk of cow, sheep, goat, camel and buffalo. In addition, we compared the *vacA* and *cagA* genotype status of *H. pylori *in human and milk samples. 

## Methods

**Sampling**

210 animal raw milk and 100 human gastric biopsy with gastrointestinal disorders were enrolled in the current study. Animal samples were collected from following species from west of Iran: cow (n=75), sheep (n=58), goat (n=42), buffalo (n=20) and camel (n=15). The samples were obtained from farm bulk tanks and milk collection centers over a year from May 2014 to March 2015 considering their Seasonality of lactating period. Sampling was performed according to the International Dairy Federation guidelines (10). Considering sterile conditions, 100 ML of each sample was collected in the sterile glass containers and transported to the laboratory at 4°C within a maximum of 3 hours after collection. 

Gastric human specimens were collected from governmental general hospital Endoscopy center in the west of Iran. The biopsy specimens were collected from antrum and corpus of each patient. Informed consent was obtained from all patients at the beginning of endoscopy. All specimens were placed in 0.1 ml of sterile saline solution, transported to the laboratory immediately and stored at -70°C until further investigation.

**DNA Analyses**

We did not apply bacterial culture methods in this investigation. We used 16S rRNA PCR analysis for the confirmation of *H. pylori *directly on all the samples. For human samples first, a rapid urease test was performed with a Gastro urease kit (Baharafshan, Iran). Then, the DNA was isolated from each biopsy, using a DNA extraction kit (CinnaGen, Iran) according to the manufacturer’s instructions and immediately used for the molecular analysis. 

 To extract DNA, 25 ml of milk samples was centrifuged at 2200 g at room temperature. Supernatant, including the hardened fat layer, was aspirated and discarded, except for the bottom 5 ml. The bottom of the remaining supernatant (including the casein pellet) was transferred by pipette into a 1±5-ml tube. To dissolve casein, 300 µl 0±5 m-disodium ethylene diamine-tetraacetate (EDTA), pH 8±0 and 200 µl 10mm-Tris-HCl±1 mm-EDTA, pH 7±6 (TE) were added to the supernatant. Somatic cells and casein micelles re-suspended by vortexing(11). Phenol-chloroform extraction and ethanol precipitation were performed, as described by Sambrook et al. (12). 

Detection of *H. pylori *was carried out in 25 μl reaction mixtures containing 2 μl ( 300 ngr) of genomic DNA, 1.5 mM MgCl2, 200 mM concentrations of dNTPs, 1 U of smar *Taq polymerase *(CinnaGen, Iran) and 0.2 mM concentrations of primers HP-1 and HP-2 under previously reported conditions. The Primers sequences were HP-1 (CTGGAGAGACTAAGCCCTCC) and HP-2 (ATTACTGACGCTGATTGTGC) (12). The applied Primers for *vacA* allels and *cagA* gene have been described previously(11). DNA samples from *H pylori *(D0008; Genekam, Germany) were used as a positive control, and sterile distilled water was used as a negative control. The PCR was performed in a DNA Thermal Cycler (Eppendrof Mastercycler 5330; Eppendorf-Nethel-Hinz GmbH, Hamburg, Germany), with 40 cycles for HP primer and 35 cycles for *vacA *and *cagA* primers. Each cycle consisted of denaturation at 95°C/45 seconds, annealing at 59°C/30 seconds for HP, 52°C/45 seconds for *vacA *and 58°C/45 seconds for *cagA *as well as extension at 72°C/45 seconds. There was another longer extension of 6 minutes at 72°C. The PCR products were visualized after electrophoresis on a 1.5% agarose gel stained with EtBr.

**Statistical analysis**

Comparisons of differences and similarities were conducted by the chi-square test using the SPSS for Windows statistical software (version 17; SPSS, Inc., Chicago, IL, USA). *P*-values less than 0.05 were considered as statistically significant. 

## Results

A total of 210 raw animal milk specimens and 100 human gastric biopsies were collected, among which 28 (13.33%) samples were confirmed to be *H. pylori *positive based on PCR assays. The frequencies were as follows: cow 12(16%), sheep 8(13.79%), goat 2 (4.76%), camel 2(13.33%), and buffalo 4(20%) (Table1). 

**Table 1:** The frequency of *H. pylori* in ruminant raw milk samples

**Species**	**Sample**	***H. pylori*** ** Pos.**
Cow	Raw milk (n=75)	12 (16.00%)
Sheep	Raw milk (n=58)	8 (13.79%)
Goat	Raw milk (n=42)	2 (4.76%)
Camel	Raw milk (n=15)	2 (13.33%)
Buffalo	Raw milk (n=20)	4 (20.00%)
Total	Raw milk (n=210)	28 (13.33%)

From the physical point of view all the ruminants seemed healthy and their milk was transported for daily consumption. Based on RUT and PCR assays, out of 100 patients, 82 (82%) patients were infected with *H. pylori, *49 individuals (49%) were men and 51 (51%) were women with an average age of 47 years (range, 17 to 87 years). Moreover, 8 (8%) of patients had gastric ulcers, 11 (11%) had duodenal ulcers, 2 (2%) had gastric cancer, 80 (80%) had gastritis, 3(3%) had duodenitis, 17(17%) had gastric nodularity and 26(26%) had gastric erosions. It should be noted that some patients had several diseases together.

The *vacA* allele types were indicated in animal samples. Among which *vacA* s1a/m1a, s1a/m2, s1a/m1a-m2, s1a/m1b-m2, s1a/m1b-m2 were the most predominant strains prospectively in sheep, cows, goats, camels and buffalos respectively. While the frequency of this gene in human were as follows: 55 (67.07%) samples s1 and 27 (32.92%) s2. Out of 67 s1 strains, 32 (39.02%) samples were s1a, 10 (12.19%) were s1b and 13(15.85%) were s1c positive. For m region, 28 (34.14%) were classified as m1 and 54 (65.85%) were classified as m2. In the case of m1 sub-typing, the distribution of m1a and m1b was 22(26.82%) and 7(7.31%) respectively. Based on table 2, the frequency of the *cagA*-positive samples were 83.33%, 50%, 100%, 50% and 75% in cows, sheep, goat, camel and buffaloes respectively however 92.68% of human *H. pylori*-positive samples harbored this gene. From the statistical point of view there was not significant relationship between *cagA* status in mentioned animals’ milk and human being (*p*=0.9). However, there was a considerable correlation between *vacA* s1a/m2 strains both in cows and sheep (*p*=0.04). Furthermore, there was a notable association between *s1b* allele in sheep and human being (*p*=0). We were not able to find any meaningful similarity in camel and buffalo with the others. 

**Table 2:** The frequency of *cagA* and *vacA* genotypes in human gastric biopsies and ruminant raw milk samples

Species	Sample positive	*cagA *positive(%)	*vacA* *s1a*(%)	*vacA* *s1b*(%)	*vacA* *s1c*(%)	*vacA* *s2*(%)	*vacA* *m1a*(%)	*vacA* *m1b*(%)	*vacA* *m2*(%)
Human being	Gastric biopsy (82)	76(92.7)	32(39)	10(12.2)	13(15.8)	33(32.9)	22(26.8)	6(7.3)	54(65.8)
Cow	Raw milk (12)	10(83.3)	9(75)	2(16.7)	1(8.3)	-	5(41.7)	-	7(58.3)
Sheep	Raw milk (8)	4(50)	4(75)	3(37.5)	-	1(12.5)	-	2(25)	6(75)
Goat	Raw milk (2)	2(100)	1(50)	1(50)	-	1(50)	-	-	2(100)
Camel	Raw milk (2)	1(50)	1(50)	-	1(50)	-	-	1(50)	1(50)
Buffalo	Raw milk (4)	3(75)	-	2(50)	-	2(50)	3(75)	-	1(25)

## Discussion

About 3 decades after the first identification and culture of *Helicobacter pylori*, the route of transmission of it to human is still controversial (4, 13, 14). In the previous study, we confirmed the presence of *H. pylori *in some animal’s gastric tissue, thus rising further hypothesizes that foodstuff contamination may play a role in spreading of the *H. pylori *infection to humans (1). According to few studies, foodstuffs, particularly milk, have been assumed as a probable source of human infection for* H. pylori *(22). Therefore, we aimed to study the prevalence of *H. pylori *in cow, sheep, goat, camel and Buffalo milk and evaluate those strains virulence factors with human beings to find whether milk is a source of transmission or not. 

 The prevalence of *H. pylori* varies all over the world. According to previous studies, the prevalence is high in most Asian countries such as China and Japan (70-50%), Eastern European, South American and Middle Eastern countries. For example, infection rate of *H. pylori *in Chile Bulgaria, Egypt, and Saudi Arabia is 73%, 61.7%, 90%, and 80% respectively. Lower prevalence belongs to the United Kingdom (13.4%), Australia (5-20%) and Switzerland (11-26%) (15, 16, 17). Iran is clustered as a risky region in the world for *H. pylori *incidence; therefore, the study of this bacterium including its prevalence, the mode of transmission, treatment and control is crucial.

The prevalence of *H. pylori *in our survey in the west of Iran was 82% in human specimens, which was higher than the other reports from Shiraz, Yazd, Ardebil and Uremia-the other cities of Iran- where the prevalence was reported 54.5%, 30.6%, 47.5% and 75.3% respectively (18, 19,20). Variation in prevalence of H. pylori may contribute to the geographical factors, which are under the influence of host, microbe and environmental effects (21).

There are several reports regarding the prevalence of *H. pylori *in household animal’s milk. For example, Quaglia et al. from Italy has been reported the prevalence of this bacterium 33%, 50% and 25.6% in sheep, cow and goat raw milk samples respectively (23). Mousavi et al. announced that 16.66% of bovines, 35% of ovine, 28% of caprin, 15% of buffalo and 13.3% of camel milk samples were infected with *H. pylori *(27). Rahimi et al. noted that this rate was 1.41%, 12.2%, 8.7%, 23.4% and 3.6% in raw bovine, ovine, caprine, buffalo and camel milks (24). We were able to detect this bacterium in 12(16%) cows, 8(13.79%) sheep, 2 (4.76%) goats, 2(13.33%) camels, and 4(20%) buffaloes, which is somehow similar to other reports. As a result of our findings, the cow and sheep milk has a higher rate of contamination compare to goat’s milk. In our previous research we did not find *H. pylori *in goats while a considerable number of cows and sheep carried *H. pylori *in their gastric tissues (1). This finding would be more important when we notice that the main source of milk consumed in Iran is from cow and sheep, thereby these finding all together may are explanation for high rate of *H. pylori *infection in a region where sheep, cow, and buffalo serve as a main source of milk for human consumption.

Detection of low numbers of *H. pylori *in goats may contribute to the following possible reasons: goat has particular natural mechanisms of resistance to this bacterium, and inhibits the systematic circulation of this bacterium throughout the body. Another reason is that some other microorganisms like *Candidatus H. bovis* may colonize the goat’s stomach and establish the extent of the resistance of goats to the super infection with *H. pylori *(1, 25). Based on our previous study, no *H. pylori *was detected in gastric samples of goats. However, detection of this bacterium in very few goat milk samples may relate to contamination or immune deficiency in selected goats. Another possibility is that some strains of *H.pylori* can infect goats.

In the next step, we analyzed two main virulence factors; *cagA* and *vacA* status in detected *H. pylori *strains. Considering positive samples for *H. pylori*, the *cagA* gene was positive in 92% of humans, 83.33% of cow, 50% of sheep, 100% of goat, 50% of camel and 75% of buffalo. In all samples the rate of the *cagA *was high, and no meaningful differences in the prevalence of *cagA* gene among any animal and human samples was found. The *cagA* was considered as a non-useful epidemiological factor at least at the current structure. 

If we substitute the *cagA* with the EPIYA motifs or the cag pathogenicity island, we would probably find a better discriminatory measurement among different strains. 

In the case of *vacA* genotypes, except to buffalo samples, s1a/m2 was the predominant strain in studied samples. This shows that similar strains of *H. pylori *are circulating among studied animals particularly sheep and cows. The s1a/m2 was also predominant genotype among human *H. pylori;* supporting the idea in which human and livestock share same *H. pylori *strains and sheep and cow play important role in *H. pylori *epidemiology. Human being *H. pylori* was more diverse in *vacA* genotype in comparison with other samples, shows that human are infected with more varied strains of *H. pylori *representing that human is a better host for this microorganisms. Some researchers believe that sheep might be infected with ‘‘human strains’’ indicating that contact with human may put sheep at risk for developing *H. pylori *infection (26). Therefore, sheep may transmit the infection to humans through the contaminated environment by feces containing *H*. *pylori. *However, it is not well known whether *H. pylori *is transmitted from animals to human or vice versa or both of them are in cycle. 

In conclusion, although human play the main role in *H. pylori *circulation among population but more likely animals and foodstuffs has an impact on *H. pylori *epidemiology*. *In our previous study, we showed that sheep might be a possible reservoir for *H. pylor*i. In the current study, in constant with prior findings we conclude that *H. pylori *could be transmitted via milk of livestock particularly sheep, cow and buffalo. Goat has a minimum role in *H. pylori *epidemiology. However, extended studies and complementary researches in other geographical regions and animals are needed to establish a precise conclusion.
